# High-throughput screening of wheat leaf dark respiration identifies significant genetic control

**DOI:** 10.1093/jxb/erae519

**Published:** 2025-02-25

**Authors:** Rubén Vicente, Alisdair R Fernie, Estefanía Uberegui

**Affiliations:** Institute of Natural Resources and Agrobiology of Salamanca (IRNASA), Spanish National Research Council (CSIC), Salamanca, Spain; Max Planck Institute of Molecular Plant Physiology, Potsdam-Golm, Germany; Institute of Natural Resources and Agrobiology of Salamanca (IRNASA), Spanish National Research Council (CSIC), Salamanca, Spain

**Keywords:** Field conditions, fluorophore system, high-throughput phenotyping, leaf dark respiration, oxygen consumption, wheat

## Abstract

This article comments on:

**Gaju O, Bloomfield KJ, Negrini ACA, Bowerman AF, Cullerne D, Posch BC, Bryant C, Fan Y, Spence M, Stone B, Gilliham M, Furbank RT, Molero G, Pogson BJ, Mathews K, Millar AH, Pearson AL, Reynolds MP, Stroeher E, Taylor NL, Turnbull MH, Atkin OK**. 2025. Accounting for the impact of genotype and environment on variation in leaf respiration of wheat in Mexico and Australia. Journal of Experimental Botany **76**, 1099–1115. https://doi.org/10.1093/jxb/erae449.

This article comments on:

**Gaju O, Bloomfield KJ, Negrini ACA, Bowerman AF, Cullerne D, Posch BC, Bryant C, Fan Y, Spence M, Stone B, Gilliham M, Furbank RT, Molero G, Pogson BJ, Mathews K, Millar AH, Pearson AL, Reynolds MP, Stroeher E, Taylor NL, Turnbull MH, Atkin OK**. 2025. Accounting for the impact of genotype and environment on variation in leaf respiration of wheat in Mexico and Australia. Journal of Experimental Botany **76**, 1099–1115. https://doi.org/10.1093/jxb/erae449.

**Leaf dark respiration (*R***_**dark**_ **) is a key process for plant growth, but its role in plant breeding has been overlooked compared with other processes such as photosynthesis. The low throughput of techniques for determining *R***_**dark**_  **has hindered its application in breeding programmes to date. Gaju *et al.* (2025) used a novel fluorophore system to detect O**_**2**_  **consumption and screen *R***_**dark**_  **in large field-grown bread wheat trials, demonstrating that there is significant genetic control on *R***_**dark**_**. These results show that the genetic variability in *R***_**dark**_  **can be exploited in breeding programmes to improve cereal yields.**

## Exploring wheat diversity by phenotyping methods under field conditions

There has been a loss of genetic diversity due to the use of a limited number of accessions during the domestication and breeding of many crops of strategic global importance, such as wheat (Katamadze *et al.*, 2023). This, alongside other causes, has contributed to a deceleration of wheat genetic advances in many regions in recent decades to rates below those predicted to be needed to ensure food security in an uncertain climatic future with a growing world population (Katamadze *et al.*, 2023). That said, with appropriate methodologies we can exploit the vast diversity of wheat, estimated at 0.8 million accessions (Langridge and Reynolds, 2021), to identify suitable germplasm to increase the genetic diversity of modern wheat cultivars and thereby improve local adaptation. Many plant functional traits (e.g. nutritional, metabolic, and physiological status) are nowadays evaluated non-invasively by using phenotyping approaches under field conditions based on, amongst others, red–green–blue (RGB), thermal, multi/hyperspectral, chlorophyll fluorescence, and light detection and ranging (LiDAR) technologies (Araus *et al.*, 2022). However, other key functional traits require laborious, time-consuming, and costly methodologies during their measurements and/or data processing, such as those used for assessing CO_2_ assimilation rates, stomatal conductance, respiration, grain quality, and root traits. In these cases, high-throughput field phenotyping still represents a major bottleneck for breeding (Araus *et al.*, 2022).

## Why should we take leaf dark respiration into account in breeding programmes to improve cereal yield and resilience?

Crop yield can be dissected as the result of three key processes: the radiation uptake throughout the crop cycle, the radiation use efficiency (RUE), and the portion of plant biomass that is used for the harvestable part of the crop (harvest index) (Araus *et al.*, 2021). Plant respiration plays a key role in the conversion of solar energy into biomass (RUE), being highly affected by environmental conditions such as temperature, drought, [CO_2_], light, and soil fertility, and the type of respiratory substrate (O’Leary *et al.*, 2019; Fan *et al.*, 2024). Respiration is a metabolic process that occurs in all plant cells and tissues in both the light (*R*_light_) and dark (*R*_dark_), and encompasses glycolysis, the oxidative pentose phosphate pathway, the tricarboxylic acid cycle, and the mitochondrial electron transport chain (Zhang and Fernie, 2018; O’Leary *et al.*, 2019; Tcherkez *et al.*, 2024). In this central process, the CO_2_ assimilated via photosynthesis is used to produce energy [ATP and NAD(P)H] and biosynthetic precursors to fuel plant metabolism, growth, survival, and agricultural productivity (O’Leary *et al.*, 2019; Schmiege *et al.*, 2023). Its relevance in cellular metabolism is due to its intimate connections with photosynthesis, photorespiration, nitrogen metabolism, cellular maintenance, redox regulation and signalling, protein turnover, and phloem loading (Zhang and Fernie, 2018; Coast *et al.*, 2019). Additionally, plant respiration is also a major contributor to the global carbon cycle, with variation in respiration rates needing to be taken into account by models that predict the impacts of climate change on ecosystem dynamics (Schmiege *et al.*, 2023; Wendering and Nikoloski, 2023).

*R*_dark_ is a major component of plant carbon budgets that, together with the availability of well-established methodologies for its measurement (see below), makes it a target for inclusion in breeding programmes focused on improving crop productivity and resilience or to monitor crop health (Coast *et al.*, 2019; Schmiege *et al.*, 2023). Indeed, the study of Gaju *et al.* (2025) proved that there is a large genotypic variability in wheat respiration, proposing the use of *R*_dark_ as a selection criterion. Hypothetically, the selection of efficient cultivars for crop improvement would be associated with a reduced rate of *R*_dark_ (Scafaro *et al.*, 2017; Gaju *et al.*, 2025).

## From low- to high-throughput: methodologies to characterize leaf dark respiration

**Fig. 1.**
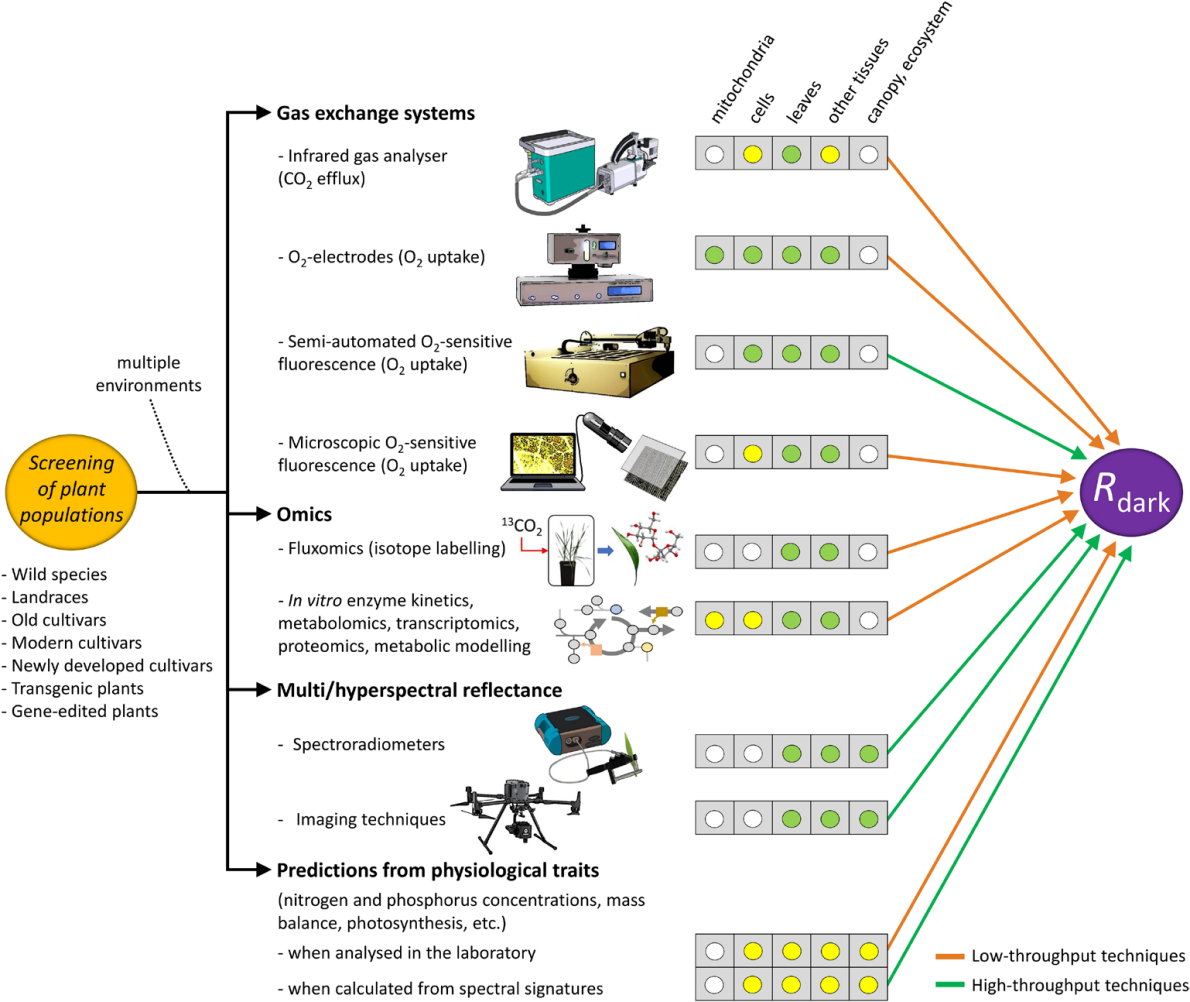
The most common approaches to estimate dark respiration (*R*_dark_) in plants. These include direct and indirect methodologies to obtain the rate of *R*_dark_, from low to high throughput, and from mitochondrial to the ecosystem level. The most common techniques for measuring *R*_dark_ include gas exchange measurements that monitor CO_2_ and O_2_ fluxes in samples using infrared gas analysers and O_2_ electrodes. These are versatile approaches that allow measurements from laboratory to field conditions, and from mitochondria to different plant organs, but are time-consuming. The fluorometric detection of O_2_ consumption allows high-throughput measurements, even under field conditions, to scale up the complexity of the experiments. Omics techniques, or those that use omics data for metabolic modelling, help to interpret respiratory metabolism in detail, but have their limitations related to the investments of both time and money. *R*_dark_ can also be estimated with high-throughput approaches based on multi- and hyperspectral reflectance at ground level or aerial level, and from other physiological parameters. While these approaches allow for an increase in the number of samples or the area to be analysed, they use predictive models that need to be improved for an accurate estimation of *R*_dark_.

Respiration is a phenotypically complex trait due to the known effects of genotype-by-environment, crop phenology, and plant tissue interactions (Scafaro *et al.*, 2017). The most used techniques to measure respiration were recently reviewed in O’Leary *et al.* (2023), listed in Fig. 1 with some modifications. Analyses of plant respiration include low-throughput techniques associated with gas exchange systems, such as CO_2_ evolution and O_2_ consumption. On the one hand, infrared gas analysers equipped with dynamic flow cuvettes (usually adapted to foliar tissues) measure the steady-state CO_2_ efflux in dark-adapted plant material as a proxy for *in vivo* respiration in natural systems (e.g. field and greenhouse experiments). This measurement usually takes from minutes to >1 h when considering response curves and the dark adaptation period. Many metabolic processes that include CO_2_-producing and -consuming steps (such as carboxylation by phospho*enol*pyruvate carboxylase, photorespiration, internal gas fluxes, etc.) interfere with gas exchange measurements, although they generally present low flux values under standard conditions (Tcherkez and Limami, 2019; Tcherkez *et al.*, 2024). On the other hand, *R*_dark_ can be determined with measurements of O_2_ consumption using closed systems, taking usually several minutes per sample (<1 h). For that, O_2_ electrodes and O_2_-sensitive luminescence quenching have been largely used for isolated mitochondria or detached tissues (Scafaro *et al.*, 2017; O’Leary *et al.*, 2023). *R*_dark_ can also be inferred from other physiological parameters (nitrogen and phosphorus concentrations, mass balance, photosynthesis, etc.), fluxomics, *in vitro* enzyme kinetics, metabolomics, transcriptomics, proteomics, or from model calculations (Coast *et al.*, 2019; O’Leary *et al.*, 2023; Wendering and Nikoloski, 2023; Tcherkez *et al.*, 2024; Gaju *et al.*, 2025) (Fig. 1), but the inferences are not always conclusive and depend on multiple factors. Among these, carbon (^13^C or ^14^C) and oxygen (^16^O_2_ or ^18^O_2_) pulse–chase labelling experiments stand out as an accurate approach for calculating respiratory metabolic fluxes (Tcherkez *et al.*, 2010; O’Leary *et al.*, 2023). However, these require significant investments of both time and money, highly trained scientists, and highly specific experimental set-ups.

**Fig. 2.**
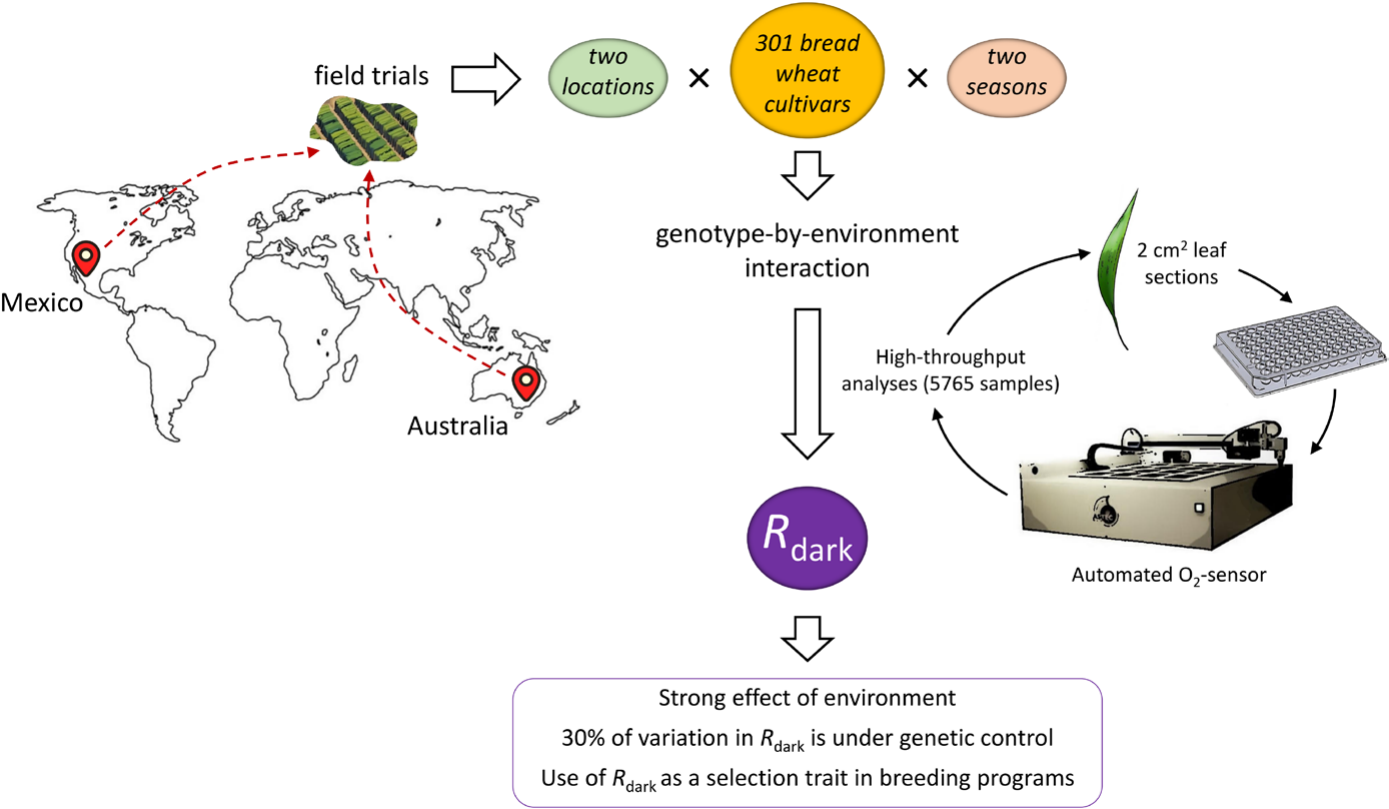
Graphical visualization of the study carried out by Gaju *et al*. (2025) to investigate the effects of genotype-by-environment interaction on dark respiration (*R*_dark_). For that, a large wheat germplasm collection was grown in Mexico and Australia during two crop seasons to analyse 5765 leaf samples by using a high-throughput system. This unprecedented study of *R*_dark_ reported large effects of environmental conditions on the variation of this trait, as well as significant genetic control, which allows proposing the use of *R*_dark_ as a selection attribute in breeding programmes to improve radiation use efficiency.

To exploit the full genetic diversity available to accelerate breeding, it is important to use high-throughput methods that quantify whether *R*_dark_ is a useful parameter for breeding and, consequently, to identify cultivars of interest to farmers and breeders in multiple environments. In this sense, Sew *et al.* (2013), Scafaro *et al.* (2017), and O’Leary *et al.* (2017) demonstrated the use of commercial sensor cartridges for the fluorometric detection of O_2_ consumption to estimate *R*_dark_ in a few minutes per sample, including long duration measurements, in Arabidopsis, eucalyptus, and wheat. This approach employs freshly detached tissues, placed in tubes in gas or liquid phases containing a fluorescent metal organic dye in the lids that is sensitive to O_2_ quenching, to detect the fluorescence signal associated with O_2_-dependent decay using an automated O_2_ sensor. The study of Gaju *et al.* (2025) is a proof-of-concept for the fluorophore system to measure *R*_dark_ in large-scale trials in crops and to explore the genotype-by-environment interaction (Fig. 2). While Scafaro *et al.* (2017) and Coast *et al.* (2019) used the same methodology in 90–138 bread wheat genotypes under controlled and varied environmental conditions, Gaju *et al.* (2025) took the experimental design to another level, studying 5765 samples, combining 301 bread wheat cultivars, two countries, and two crop seasons. This is the largest dataset of leaf *R*_dark_ in wheat, with a meticulous experimental design to reduce residual variation to a minimum (<10%, compared with 20–75% in previous studies) in order to properly assess the genotype variability and environmental effects. The results showed a high variation (16-fold range) of temperature-normalized rates of *R*_dark_ when expressed on an area basis, being a third of the variation under genetic control. This lays the groundwork for identifying the genetic basis for its variation using quantitative trait loci mapping populations and genome-wide association studies (Bulut *et al.*, 2023). It also highlights that there is room to use *R*_dark_ as a novel trait for improving RUE and hence wheat grain yield. Moreover, half of the total variation in *R*_dark_ was under environmental control, as would be expected, considering plot position and growth conditions (field location, crop season, and day-to-day variability) (Gaju *et al.*, 2025). Crop phenology influences the *R*_dark_ rate and its regulation, so it is important to monitor phenology to measure it at common growth stages for a population (although daily fluctuations in temperature can have a significant impact) or obtain high-resolution phenotyping data during time-series (Zhang and Fernie, 2018; Araus *et al.*, 2022). The main limitations of the fluorophore system include sample destruction and restricted comparison between studies using other technologies. However, the scarcity of results in this field, the high-throughput nature of this approach, and the vast number of plant accessions available guarantee the expansion of these studies in the next years.

The possibility to measure *R*_dark_ on a large number of samples, unlike using traditional gas exchange methods, could help to develop more robust models to define spectral reflectance indices that take *R*_dark_ measurement to an even larger scale (Coast *et al.*, 2019; Gaju *et al.*, 2025). Spectral measurements are extremely fast (in the range of seconds when using spectroradiometers) and non-destructive, although to date current models in wheat can still be improved (*r*^2^ values of 0.50–0.63 and poor model performance across experiments) (Coast *et al.*, 2019). Hyperspectral imaging offers the advantage of phenotyping large areas (using drones or satellites), but these methods have a more limited spectral range and data processing is more challenging. With a similar fluorescent optical sensor foil and a USB microscope, Tschiersch *et al.* (2012) established a pipeline for a microscopic imaging-based approach to measure O_2_ consumption (respiration) or evolution (photosynthesis). This alternative approach provided fascinating spatial dynamics of respiration in heterogeneous plant tissues, but its scalability is limited. Whether measured by the fluorophore system or by hyperspectral reflectance, it is clear that *R*_dark_ has taken on a new dimension in recent years that allows its integration into multi-dimensional datasets and breeding programmes as a novel tool for selection.

## Future prospects

With the knowledge that *R*_dark_ is under genetic control in wheat (Gaju *et al.*, 2025), it will be worth assessing the reliability of the high-throughput methodology proposed on other crop species in multiple environments (treatments, crop management practices, locations, etc.). Moreover, the spatial–temporal application of the fluorophore system for *R*_dark_ remains to be evaluated (i) throughout developmental stages to identify the best periods to predict complex traits (such as yield), (ii) for its scalability to higher levels or difficult-to-measure units (i.e. tissues, organs, whole plants, canopies, ecosystems, and global climate change modelling), and (iii) for metabolic engineering focused on improving carbon-use efficiency, grain yield, and crop resilience. Finally, future studies may further define spectral indices and identify genomic regions associated with *R*_dark_ that contribute to large-scale phenotyping, marker-assisted breeding, and gene editing approaches.
